# Editorial: Antimicrobials alternatives for the prevention and treatment of veterinary infectious diseases

**DOI:** 10.3389/fvets.2022.1025150

**Published:** 2022-09-09

**Authors:** Muhammad Fakhar-e-Alam Kulyar, Xiushuang Chen, Zeeshan Ahmad Bhutta, Prerona Boruah, Samina Shabbir, Muhammad Akhtar, Amjad Islam Aqib, Ambreen Ashar, Kun Li

**Affiliations:** ^1^Institute of Traditional Chinese Veterinary Medicine, College of Veterinary Medicine, Nanjing Agricultural University, Nanjing, China; ^2^College of Veterinary Medicine, Huazhong Agricultural University, Wuhan, China; ^3^Laboratory of Biochemistry and Immunology, College of Veterinary Medicine, Chungbuk National University, Cheongju, South Korea; ^4^DY Patil, Deemed to be University, Navi Mumbai, Maharastra, India; ^5^Institute of Plant Protection, Guangdong Academy of Agricultural Sciences, Guangzhou, China; ^6^Department of Medicine, Cholistan University of Veterinary and Animal Sciences, Bahawalpur, Pakistan; ^7^Department of Chemistry, Government College Women University, Faisalabad, Pakistan

**Keywords:** antimicrobials, antibiotics, antibiotic alternatives, veterinary, infectious diseases, veterinary infectious diseases, probiotics, antimicrobial peptides

Antibiotics have carried the promise of curing and managing infectious diseases throughout their discovery, resulting an enormous increase in antibiotic use in all sectors, including routine animal husbandry techniques (1). Laws against the extra-label use of antibiotics are uncommon, even in developed countries. So, the rate of rising in the prevalence of antimicrobial resistant infections is now inversely related to the rate at which new medications are being approved. Therefore, developing resistance to antimicrobials is one of the most pressing public health issue of the 21st century. When veterinary medicine is considered, the quantity of antibiotics utilized by animals is almost twice that used by humans (2). Long periods have been spent using such extensive antimicrobial medications to either increase feed conversion or avoid diseases. These non-therapeutic applications allow an extraordinary selection pressure on bacteria, resulting in novel survival strategies. These adaptation strategies are responsible for antibiotic resistance. According to the most recent findings in scientific research, this phenomenon seems to be wired, although it was only contextualized after the conclusion of World War II. Its goal was to enhance animal protein production *via* low-cost methods, a goal still relevant today in countries with low per capita incomes (3). In such countries, the use of antimicrobials in animal feeding as growth promoters is highly linked with the development and spread of antimicrobial-resistant, which makes it difficult to cure infectious diseases.

Antibiotics are administered to many food-producing animals to reduce the risk of infections and compensate for the lack of sanitary conditions on commercial livestock farms (4). Therefore, livestock serves as a source that disperses resistant bacteria into the environment *via* the consumption of contaminated bio wastes, milk, or meat, as well as through direct contact with animals and people who work on animal farms. Similarly, zoonotic diseases may transfer their antibiotic resistance to humans. Because animals and commodities derived from livestock are carried throughout the globe. In same sense, animal and plant diseases that disrupt the food supply in one country might spread to other countries and cause problems elsewhere. In addition, germs may become resistant to antibiotics when they are subjected to low concentrations over extended periods. Feeding livestock antibiotics at low doses is a widespread technique used to increase animal body weight. These antibiotics are also administered arbitrarily to avoid diseases in herds or flocks that have restricted space. These kinds of behaviors help to foster the development of antibiotic resistance as well as its subsequent dissemination. The methods result a significant buildup of antibiotics in the environment and the development of antibiotic resistance in the microorganisms that come into contact with them (5). However, it is difficult to identify the route and capability of resistance elements from animals to people and to establish that animals serve as reservoirs of resistant genes since it is difficult to understand how resistance factors are passed between the species. The fact that there are naturally occurring resistance genes in the environment adds a layer of complexity to the situation. For instance, it was discovered that natural resistance to antibiotics occurred a very long time before the development of agriculture (6).

Several options other than traditional antibiotics have been developed to tackle antimicrobial resistance and manage bacterial infections. Some methods have taken a step while others are still in the research phase. However, the quick adoption of alternatives is hampered by several issues. Experts agree that a shortage of governmental and private funds for antibiotics' alternative research is the most pressing problem. The low level of interest in this field is often linked to the hope of confirming the efficacy of products that have already undergone preliminary testing. In addition to the product's qualities, its popularity may also be attributed to the openness of farmers and veterinarians to new possibilities. Since veterinarians play such a pivotal role in raising farmers' understanding of animal health and the principles of new treatments. Such novel options must have a clear mechanism of action, and their use must be simple and obvious to both the veterinarian and the farmer (7). As a result, there is a need for a multi-actor, cross-sectoral strategy to address animal diseases. Furthermore, the effectiveness, toxicity, and mechanism of these therapies should be investigated during pre-clinical trials to optimize their dose and formulation. The findings and a survey of veterinarians, farmers, and consumers' perceptions of antimicrobial alternatives should be used to identify treatments for large-scale farm trials, i.e., measuring clinical effectiveness and effects on antimicrobial usage. Furthermore, a portfolio of numerous options for treating or preventing infectious diseases, such as phage treatment, antimicrobial peptides, bacteriocins, probiotics, fecal transplant therapy, predatory bacteria, and antibodies, should be developed (Figure 1).

**Figure 1:**
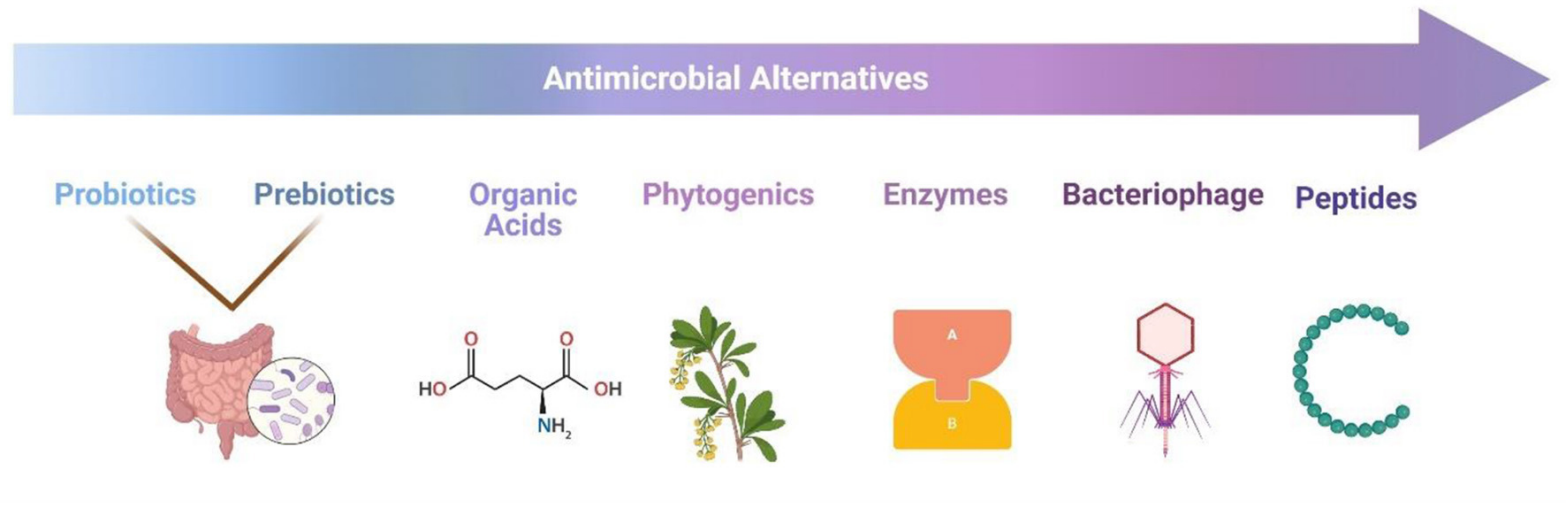
Various options for antimicrobial alternatives that are normally available all over the world.

Meanwhile, we must remember that “prevention is better than cure.” Antibiotics are still useful in preventing and managing animal infections in many underdeveloped countries due to the poor agricultural environment and high disease incidence. It is shown that the ban on growth promoters necessitates increased agricultural hygiene. When the number of “old” antibiotics used in feed decreases due to the ban, the frequency of bacterial infections is expected to grow unless the production environment is fundamentally improved. This may increase the therapeutic use of powerful antibiotics, which may have unforeseen repercussions for public health problems. Furthermore, there is no scientific evidence to distinguish the causal link between antibiotic therapy and preventative usage regarding resistance development. Before enacting such a strategy due to political/social pressure, the final benefit and danger should be examined since microorganisms may not always “listen” to politicians. As a result, the choice to administer in-feed antibiotics should be founded on scientific evidence. Antibiotic bans as growth promoters cannot be repeated in every country. Some “vintage” medicines may identify new bacterial targets and boost anti-infectious treatment for certain bacteria. Also, novel formulations may enable targeted drug administration, and increase the interaction of molecules through antibacterial activity (8). Hence, it is essential to switch to the most appropriate narrow-spectrum agent as soon as an infection has been brought under control and the culture and susceptibility testing results have been reported. Doing so reduces the potential for adverse drug reactions and the chance of developing antibiotic-induced resistance.

In summary, it is essential to find a suitable replacement for antibiotics with the improvement of animal feed administration, production, and hygiene. In addition to conducting research and developing new alternatives that are efficient and risk-free, we should investigate the possibility of combining the application of antibiotics and their alternatives in order to sustain a healthy agricultural economy and ensure their potential for use in the veterinary sector.

## Author contributions

MK and XC wrote the first draft of the manuscript. All other authors have made a substantial, direct, and intellectual contribution to the work and approved it for publication.

## Funding

This study was supported by the Start-up fund of Nanjing Agricultural University (804131) and the Start-up Fund for Distinguished Scholars of Nanjing Agricultural University (80900219).

## Conflict of interest

The authors declare that the research was conducted in the absence of any commercial or financial relationships that could be construed as a potential conflict of interest.

## Publisher's note

All claims expressed in this article are solely those of the authors and do not necessarily represent those of their affiliated organizations, or those of the publisher, the editors and the reviewers. Any product that may be evaluated in this article, or claim that may be made by its manufacturer, is not guaranteed or endorsed by the publisher.
